# LLM-Assisted Interpretation of Kinematic Gait Data in Children with Cerebral Palsy: A Pilot Study on Gait Deviation Detection and Surgical Group Recommendations

**DOI:** 10.3390/bioengineering13080862

**Published:** 2026-07-25

**Authors:** Mehrdad Davoudi, Jacqueline Romkes, Michèle Widmer, Chris Easthope Awai, Elke Viehweger

**Affiliations:** 1 Neuroorthopaedics and Centre for Clinical Motion Analysis, Department of Orthopaedics, University Children’s Hospital Basel, 4056 Basel, Switzerland; j.romkes@unibas.ch (J.R.); michele.widmer@ukbb.ch (M.W.); heide.viehweger@ukbb.ch (E.V.); 2 Clinic for Orthopaedics, Heidelberg University Hospital, Schlierbacher Landstr. 200a, 69118 Heidelberg, Germany; 3 Data Analytics & Rehabilitation Technology (DART), Lake Lucerne Institute, 6534 Vitznau, Switzerland; chris.awai@llui.org; 4 Department of Health Sciences & Technology, ETH Zurich, 8093 Zurich, Switzerland; 5 Department of Biomedical Engineering (DBE), University of Basel, 4123 Allschwil, Switzerland

**Keywords:** cerebral palsy, gait analysis, large language model, clinical decision support

## Abstract

Three-dimensional instrumented gait analysis is widely used to guide surgical decision-making in children with cerebral palsy (CP), but its interpretation is time-consuming and prone to inter-rater variability. In this single-centre pilot study, we investigated whether a generative large language model (LLM) could consistently generate gait deviation findings and surgical procedure suggestions that align with expert judgement. Kinematic features for lower-limb joints across the gait cycle, stance, and swing were extracted from eight children with unilateral CP using the open-source GaitSharing Toolkit and a structured prompt, then submitted three times per patient to OpenAI’s GPT-5.5 model. The model assessed 28 kinematic deviations and 12 surgical procedure groups using majority voting. One gait analyst and two paediatric orthopaedic surgeons independently rated outputs on a 0–2 ordinal scale, blinded to all clinical information beyond the kinematic curves and diagnosis. Agreement was summarised descriptively as the percentage of the maximum attainable score with 95% confidence intervals (CIs), and quadratic-weighted Cohen’s kappa was used to quantify inter-surgeon agreement. Agreement with the gait expert was highest at the hip (90.6%) and lowest at the knee, particularly in the transverse plane (65.2%). For surgical procedures, agreement with the LLM reached 83.9% and 73.4% for the two surgeons, with the tibialis anterior procedure showing the lowest concordance. Inter-surgeon agreement was 79.2% (95% CI 71.9–85.4) with a kappa of 0.59 (0.47–0.70), indicating moderate agreement. The LLM showed high self-consistency (>90% across runs). These preliminary findings suggest that generative LLMs may be feasible as assistive tools in clinical gait analysis for deviation detection and future treatment planning and should be interpreted as hypothesis-generating, warranting confirmation in larger, more diverse cohorts.

## 1. Introduction

Cerebral palsy (CP) is the most common cause of physical disability in childhood, with a substantial impact on overall development, particularly on walking ability [1,2]. The global prevalence of CP is estimated to range from 1.6 to 3.3 per 1000 live births [3]. Although no curative treatment currently exists, a combination of surgical and conservative interventions is used to optimize physical function [1]. In ambulatory children with CP, three-dimensional instrumented gait analysis (3D-IGA) plays a central role in clinical decision-making, especially when surgical intervention is considered [4]. A single 3D-IGA assessment yields hundreds of kinematic, kinetic, electromyographic, and spatiotemporal variables, which must be integrated with clinical findings, imaging results, and patient history. This synthesis imposes a considerable cognitive burden, and interpretation is widely recognized to depend strongly on the experience and clinical judgment of the treating clinician. The inter-observer reliability of this interpretive process has been extensively investigated in the literature [5,6,7] suggesting that while the interpretation of gait analysis outcomes is reproducible in principle, clinically meaningful variability persists across institutions, individual clinicians, and over time. In the context of gait with CP, particularly in the presence of multi-level involvement and complex severity, this process may lead to inconsistent clinical judgment and less predictable outcomes [8].

In recent years, machine learning approaches have increasingly been applied to gait analysis data, including electromyography (EMG) and clinical examination parameters, to develop objective artificial intelligence (AI)-driven tools that support more informed clinical decision-making in children with CP [9,10,11]. Despite these advances, such evidence-informed decision-making frameworks have not been widely integrated into routine clinical practice, potentially due to the lack of accessible platforms capable of consolidating and operationalizing these tools. To address this challenge, Schwartz et al. introduced the Evidence-Based Gait Analysis Interpretation Tools (EB-GAIT) framework [8], which employs Bayesian models trained to estimate the probability of commonly performed surgical procedures. However, current clinical AI tools, including EB-GAIT, rely on predefined feature sets and structured inputs rather than generating explicit clinical reasoning.

Large language models (LLMs), including Chat Generative Pre-trained Transformer (ChatGPT), have recently demonstrated substantial potential for complex clinical reasoning across a wide range of medical domains [12,13,14,15]. While the use of LLMs for gait analysis, particularly for gait pattern classification across different pathologies, has already been explored [16,17,18,19], these models have not yet been systematically assessed for interpreting kinematic gait data and decision-making in CP. Whether such models can reliably replicate expert clinical reasoning remains an open and clinically important question, which this study aims to investigate.

To investigate this, we evaluated whether a general-purpose LLM, provided exclusively with structured kinematic features, could detect individual gait deviations from reference norms and reason about corresponding surgical procedure recommendations. The LLM decision space was constrained to a subset of clinically informed deviations and interventions derived from the literature [6,7,8].

As an early-stage, single-centre pilot study, our aim was not to confirm performance against a pre-specified benchmark but to characterise it descriptively and identify where such a system may, or may not, be clinically useful. We therefore pursued four exploratory objectives: (i) to assess overall agreement between LLM outputs and expert judgement for both gait deviation detection and surgical procedure recommendations; (ii) to describe how this agreement varies across planes of motion, anatomical levels, and surgical procedure groups; (iii) to examine inter-rater agreement among surgeons reviewing the same LLM-generated recommendations, as an indicator of how clinical factors shape decision-making even within a single centre; and (iv) to quantify the internal replication confidence (self-consistency) of the LLM across repeated runs. All analyses are descriptive and hypothesis-generating, and findings should be interpreted as a basis for adequately powered confirmatory work rather than as established performance estimates.

## 2. Materials and Methods

### 2.1. Ethics Statement

This study was approved by the local ethics committee (“Ethikkommission Nordwest- und Zentralschweiz, EKNZ Nr. 2025-01762”). Written informed consent was obtained from all participants or, in the case of minors under the age of 14 years, from their parents or legal guardians. To ensure data privacy, only derived kinematic features and a simplified diagnostic descriptor were transmitted to the external LLM service. No patient-identifiable information, including name, surname, age, sex, clinical history, or disease severity, was shared. Furthermore, full kinematic waveforms, spatiotemporal parameters, kinetic data, EMG, video recordings, medical imaging, or clinical examination notes were not transmitted at any stage of the pipeline. The output generated in this study was not used for clinical decision-making and did not influence the treatment recommendations of the participating surgeons. The surgeons were blinded to patient identity throughout the evaluation process. Access to the study data (C3D files) was obtained on May 7, 2026, by selecting eligible patients (referred to also as “cases” throughout the manuscript) based on anonymised gait laboratory identifiers. No prior knowledge of the patients’ underlying gait abnormality was involved in the selection process.

### 2.2. Participants

This study included clinical gait data from eight patients with unilateral CP and 20 typically developing (TD) individuals, all aged between 7 and 17 years. Only the affected side was included in subsequent analyses. Inclusion criteria for all participants were the availability of complete kinematic data and the ability to walk barefoot without assistive devices. The patients were classified as Gross Motor Function Classification System (GMFCS) level I (six patients) or II (two patients). Only patients with written consent were included. The TD cohort has been described in our previously published works [20,21]. Individuals with any known neurological or orthopaedic impairments, a history of major lower limb surgery, or a leg length discrepancy greater than 1% of body height were excluded. Demographic characteristics of both CP and TD participants are summarized in Table 1.

### 2.3. Experimental Protocol

Data collection was conducted in the movement laboratory at the University Children’s Hospital Basel (UKBB, Switzerland). Participants walked barefoot at a self-selected speed over a minimum distance of 6 m, with one practice trial allowed prior to recording. Kinematic data were collected using a 12-camera motion capture system (Vicon, Oxford Metrics Limited, Oxford, UK) at 150 Hz, with reflective skin-mounted markers placed according to the protocol described by Kadaba et al. [22] and the Plug-in-Gait (PiG) model used for analysis. Seven valid trials were obtained per participant; trials containing tracking errors, missing markers, or non-steady walking patterns were excluded. Gait cycles were defined by visually identifying foot strike and toe-off events, and marker trajectories were filtered using the built-in Woltring filter in Vicon Nexus (Version 2.9.3). Joint kinematics were calculated using the Conventional Gait Model (CGM 1.0, Plug-in-Gait, Vicon Nexus, UK) [23], with body segment inertial parameters based on Dumas et al. [24]. Joint angles for the pelvis, hip, knee, and ankle were extracted in the sagittal, frontal, and transverse planes, and the foot progression angle was computed as an indicator of in-toeing or out-toeing gait. All data were stored in C3D format. For the TD cohort, 39 trials comprising 155 strides were used to establish reference kinematic values. For the CP group, 53 trials from eight patients yielding 131 strides were analyzed, with interpretation restricted to the clinically affected side.

### 2.4. GaitSharing Toolkit

All processing steps, from raw C3D files to the final LLM-generated report, were performed using GaitSharing, a freely available open-source toolkit previously developed in Python (version 3.13) by the authors. The toolkit is publicly accessible at [25,26]. It implements a fully integrated, end-to-end pipeline composed of the modules described below, each of which is accessible via a graphical user interface. The C3D Extractor module imports preprocessed and modeled kinematics data using the ezc3d library [27], and segments strides for both right and left sides using heel-strike and toe-off events recorded in the C3D. The strides were time-normalised to 101 data points using linear interpolation (interp1d function). The stance phase proportion was calculated to enable subsequent labeling of stance and swing phases. This was followed by a feature extraction stage to transform each kinematic curve into a compact tabulated representation as an input to the LLM. For each joint (pelvis, hip, knee, ankle, and foot progression angle) and plane of motion (sagittal, frontal, transverse), six summary features were extracted: max, min, their timing within the gait cycle, mean, and range, recommended by [9,10,28]. These are calculated over the gait cycle, as well as for the stance, and swing phases on both sides, first at the stride level and then averaged across all valid strides for each patient. A dataset of TD individuals (described in Section 2.2) was processed through the same pipeline to establish reference values. All the features exported to LLM were paired with a corresponding TD value to further facilitate identification of deviations.

### 2.5. LLM Interpretation

The LLM Interpreter module of GaitSharing communicates with the OpenAI Application Programming Interface (API) using a user-provided API key. The model employed in this study was gpt-5.5-2026-04-23. All API calls were performed within a single session, with each assessment requiring less than five minutes on a standard gait laboratory workstation (Intel Core i7-8700, 16 GB RAM). For traceability and reproducibility, the toolkit automatically logged the model version, execution timestamp, patient identifier, and diagnostic context for each run in a local text file. Sampling parameters (temperature and top_p) were left at their API default values, and no random seed was specified, so the three runs per patient reflect the model’s natural stochasticity rather than a tuned configuration.

Prior to submission, all input files were anonymised to remove patient-identifiable information. File names were replaced with a generic descriptor (“patient_kinematics.txt”), and the diagnostic context provided to the model was simplified to its essential form: “unilateral cerebral palsy with affected right/left side.” A standardized prompt was used for all patients to ensure consistency in reasoning and output structure. The prompt instructed the model to assume the role of a paediatric orthopaedic surgeon and gait analyst specializing in cerebral palsy, with reasoning strictly limited to the kinematic features and diagnostic descriptor.

Phase 1- Gait deviation detection: The model assesses a predefined list of 28 kinematic gait deviations on the affected side only (Table 2) [6,7,8]. For each deviation, the model responds YES, NO, NMI (need more information) or INC (inconclusive). When responding YES, it must record the exact variable name and value from the input file as supporting evidence.

Phase 2- Surgical procedure suggestions: Based exclusively on the deviations marked YES in Phase 1, the model evaluates twelve standard procedure groups (Table 3) [6,7,8], and responds with the same types as deviations. When responding YES or NMI, it must list the driving deviation numbers from Phase 1 together with a short reasoning for decision. Considering NMI as a possible response for both phases 1 and 2, provided us with this possibility to further evaluate the LLM’s reasoning from a clinical point of view. The prompt clearly specified that NMI could be assigned when data were missing or considered ambiguous (Phase 1) or when a relevant deviation was identified but further examination data were needed to confirm the decision (Phase 2). An example of an NMI rationale for calf muscle lengthening extracted from LLM is as follows (ROM: range of notion):

*“Deviations 23, 24—Swing plantarflexion with reduced ankle sagittal ROM may reflect dynamic equinus or dorsiflexor weakness, requiring passive ankle examination and selective motor assessment.”*

The deviation and procedure lists were derived from the existing literature, particularly references [6,7,8]. The full prompt text (StudyPrompt.txt), along with an example LLM input (LLMInput_example.txt; kinematic features) and output (LLMOutput_example.xlsx; procedure recommendation), are provided in the Supplementary Material.

### 2.6. Expert Review

Three independent clinical experts reviewed the anonymised gait curves. The 28 kinematic deviations (Phase 1) were rated by an experienced gait analyst with more than 25 years of experience in clinical gait analysis (J.R.). The 12 surgical procedure groups (Phase 2) were rated independently by two paediatric orthopaedic surgeons: a senior surgeon with over 25 years of experience in neuro-orthopaedic surgery and gait analysis (E.V.), and an experienced paediatric orthopaedic surgeon with five years of clinical practice (M.W.). All reviewers received only the kinematic curves and the diagnostic descriptor (unilateral CP with the affected side). Reviewers were first asked to predict independently, then looking at each LLM output, rate the decision on a three-level ordinal scale: 2 appropriate (correct decision with valid reasoning); 1 partially appropriate (correct direction but flawed reasoning, or borderline); 0 inappropriate (wrong decision or evidence). The reviewers were also asked to complete a free-text commentary summarizing their assessment.

### 2.7. Statistical Analysis

For Phase 1 (gait deviation detection), agreement between the gait expert and the LLM was computed for each kinematic deviation and then aggregated across anatomical levels (pelvis, hip, knee, ankle, and foot) and planes of motion (sagittal, frontal, and transverse). When all of an item’s LLM decisions were rated 2, agreement for that item was set to 100%; otherwise, agreement was the sum of the assigned scores expressed as a percentage of the maximum attainable score (i.e., all ratings equal to 2). For example, the pelvis includes five possible deviations (Table 2). If the examiner completely agrees with all five LLM decisions, each decision receives the maximum score of 2, resulting in a total score of 5 × 2 = 10. Since 10 is the maximum possible score, the agreement ratio is 100%. In contrast, if the examiner fully agrees with two decisions and fully disagrees with the remaining three, the total score would be (2 × 2) + (3 × 0) = 4. Dividing this by the maximum possible score of 10 yields an agreement ratio of 40%.

The same metric was applied to each procedure group in Phase 2 (surgical procedure suggestions) across all patients. Inter-surgeon agreement was computed per patient as the percentage of LLM decisions for which both surgeons assigned an identical rating (0, 1, or 2), and then averaged across decisions.

To quantify the uncertainty of these estimates given the limited sample, 95% confidence intervals (CIs) were obtained using a non-parametric cluster bootstrap in which the eight patients were resampled with replacement. Resampling at the patient level preserves the correlation among items rated within the same patient. Where all available ratings 100%, the bootstrap interval was reported as such. In addition, the inter-surgeon agreement between the two surgeons was additionally summarised as a quadratic-weighted Cohen’s Kappa [29] computed across all 96 decisions (12 procedures × 8 patients). All computations were performed in MATLAB (The MathWorks, Natick, MA, USA, Version: R2023a).

### 2.8. LLM Self-Consistency Analysis

To assess the internal replication confidence of the LLM outputs, each patient’s feature file was evaluated three times under identical conditions using the OpenAI API. Each run produced an independent set of YES/NO/NMI decisions covering all 28 deviations and 12 procedure groups. If at least two of the three runs returned the same decision, that decision was considered the final LLM output. For example, a distribution of YES: 2/3 and NO: 1/3 resulted in a final decision of YES. If all three runs produced different decisions, with one YES, one NO, and one NMI, the item was classified as inconclusive (INC).

For each patient, the proportion of items with complete agreement (3/3 = 100%) and majority agreement (2/3 = 66%) was calculated separately for deviations and procedures. Overall, the LLM generated 320 decisions across the cohort: 28 deviations plus 12 procedures across 8 cases. Each decision was derived from three independent runs on identical input data. The three runs per patient were performed as fully independent API calls. For each run, the message payload was reconstructed, and no conversation history was retained. Therefore, the model had no access to its previous responses when generating subsequent outputs.

## 3. Results

**Figure 1:**
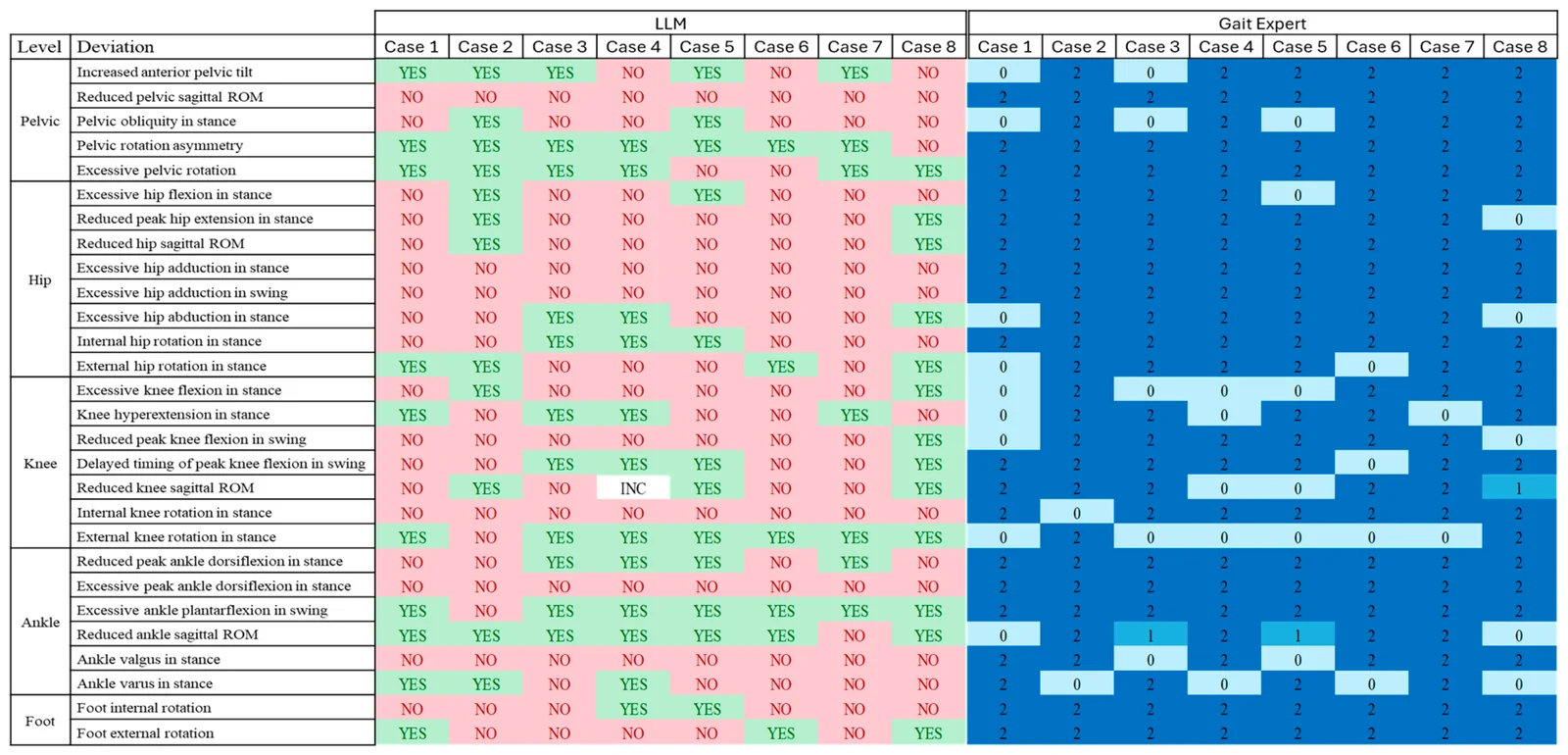
Detailed overview of gait deviations identified by the LLM and their corresponding evaluation by the expert. LLM outputs are categorized as YES (green), NO (red), NMI (need more information), and INC (inconclusive, no colour). Evaluation scores are defined as 2 (appropriate), 1 (partially appropriate), and 0 (inappropriate), represented by a colour gradient from dark to light blue.

Figure 1 presents the gait deviations identified by the LLM and their corresponding agreement with the expert across all patients. The LLM outputs were categorized as YES, NO, and, in one case, INC. A summary of the results is provided in Table 4, while the detailed data are available in DetailedTable_Figure1.xlsx in the Supplementary Material.

Agreement with the gait expert was high in the sagittal plane across most joints, with values of 87.5% (95% CI 68.8–100), 91.7% (79.2–100), 68.8% (55–82.5), and 90.6% (82.8–96.9) for the pelvis, hip, knee, and ankle, respectively. In contrast, the lowest agreement was observed for knee internal/external rotation (56.3%, 50–68.8), and for pelvic obliquity and ankle varus/valgus (both 62.5%; CI: 25–87.5 and 50–81.3, respectively). The primary concerns raised by the gait expert regarding the LLM outputs and the underlying evaluation process are summarized in Table 5, and closely align with the discrepancies observed in Table 4. A key recommendation was to consider a threshold for defining a clinically meaningful difference from TD in deviation assessment, particularly for the assessment of knee internal/external rotation. The expert also highlighted the need to include a bilateral assessment of pelvic obliquity and to revise the terminology used for ankle varus/valgus and foot rotation.

**Figure 2:**
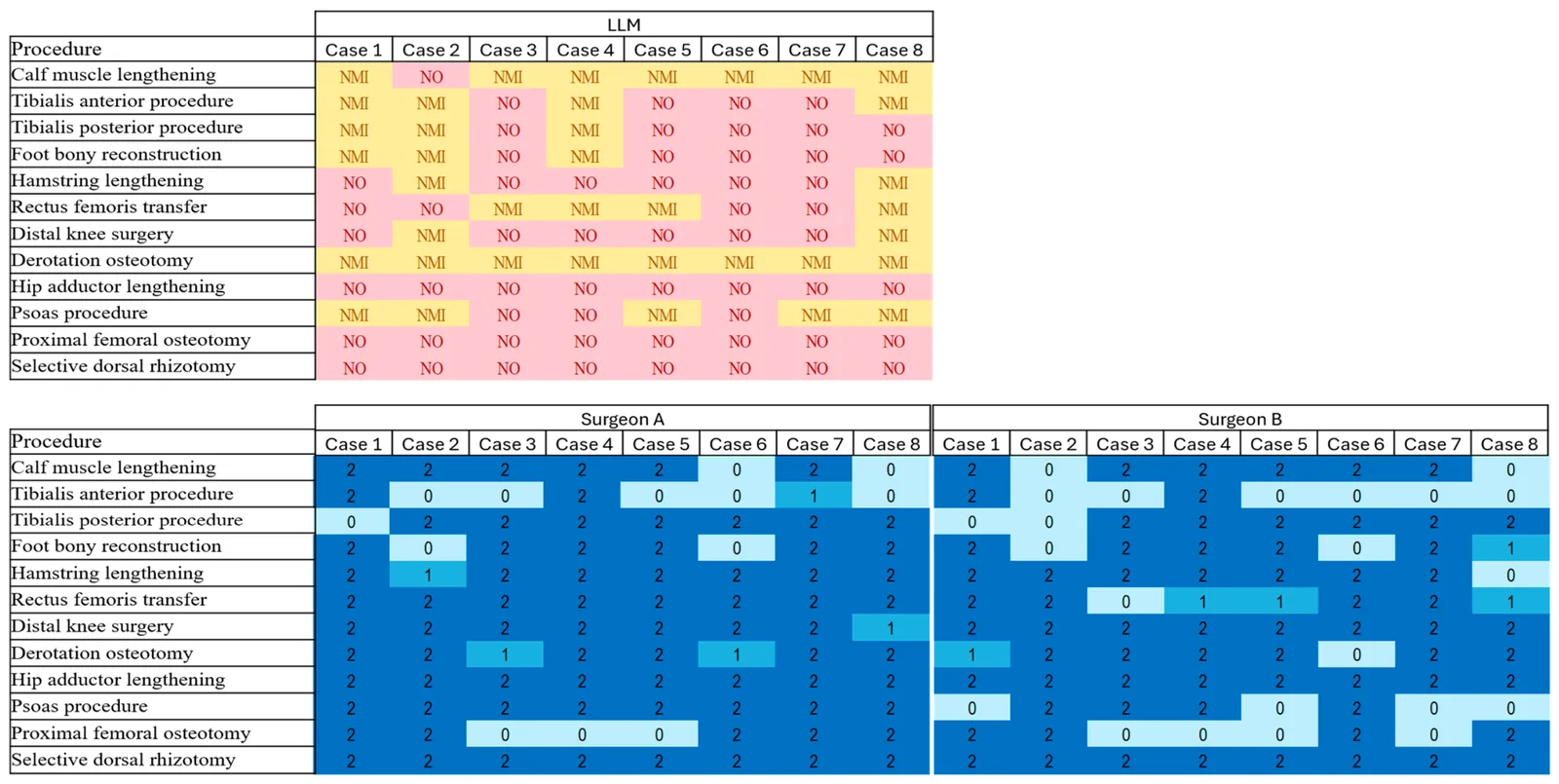
Overview of procedure groups suggested by the LLM and their corresponding evaluation by two orthopaedic surgeons. LLM outputs are categorized as YES, NO (red), NMI (need more information, yellow), and INC (inconclusive). Evaluation scores are defined as 2 (appropriate), 1 (partially appropriate), and 0 (inappropriate), represented by a colour gradient from dark to light blue.

To further evaluate the performance of the LLM across procedure groups, detailed outputs and their corresponding agreement rates with both surgeons are presented in Figure 2. Notably, the LLM did not produce any “YES” outputs, suggesting that the model appropriately identified “NMI” as a valid outcome when the available data were insufficient and thereby avoided definitive decisions based on incomplete information. The overall agreement between the surgeons and the LLM was 78.7%, with 83.9% (95% CI 78.1–89.1) for Surgeon A and 73.4% (67.7–79.2) for Surgeon B (Table 6). The lowest levels of agreement were observed for the tibialis anterior procedure (31.3%, 6.3–62.5 for Surgeon A; 25%, 0–50.3 for Surgeon B) and proximal femoral osteotomy (62.5%, 25–87.5 for Surgeon A; 50%, 12.5–87.5 for Surgeon B). The width of the confidence intervals for these per-procedure estimates reflects the small number of cases and indicates that they should be regarded as preliminary signals rather than precise performance values.

Despite these discrepancies, inter-surgeon agreement remained moderate to high, averaging 79.2% (95% CI 71.9–85.4) across cases with a quadratic-weighted Cohen’s kappa of 0.59 (0.47–0.7). The lowest value was observed for patient 8, where agreement dropped to 58.3% (Table 7). Both surgeons emphasized the need for additional clinical examination data to support precise decision-making (Table 8). Surgeon A more frequently considered combined procedures such as tendo-Achilles lengthening with tibialis anterior tendon shortening (TAL-TATS) for several patients, whereas Surgeon B highlighted the importance of patient age and the potential compensatory role of the contralateral limb in guiding treatment decisions.

**Figure 3:**
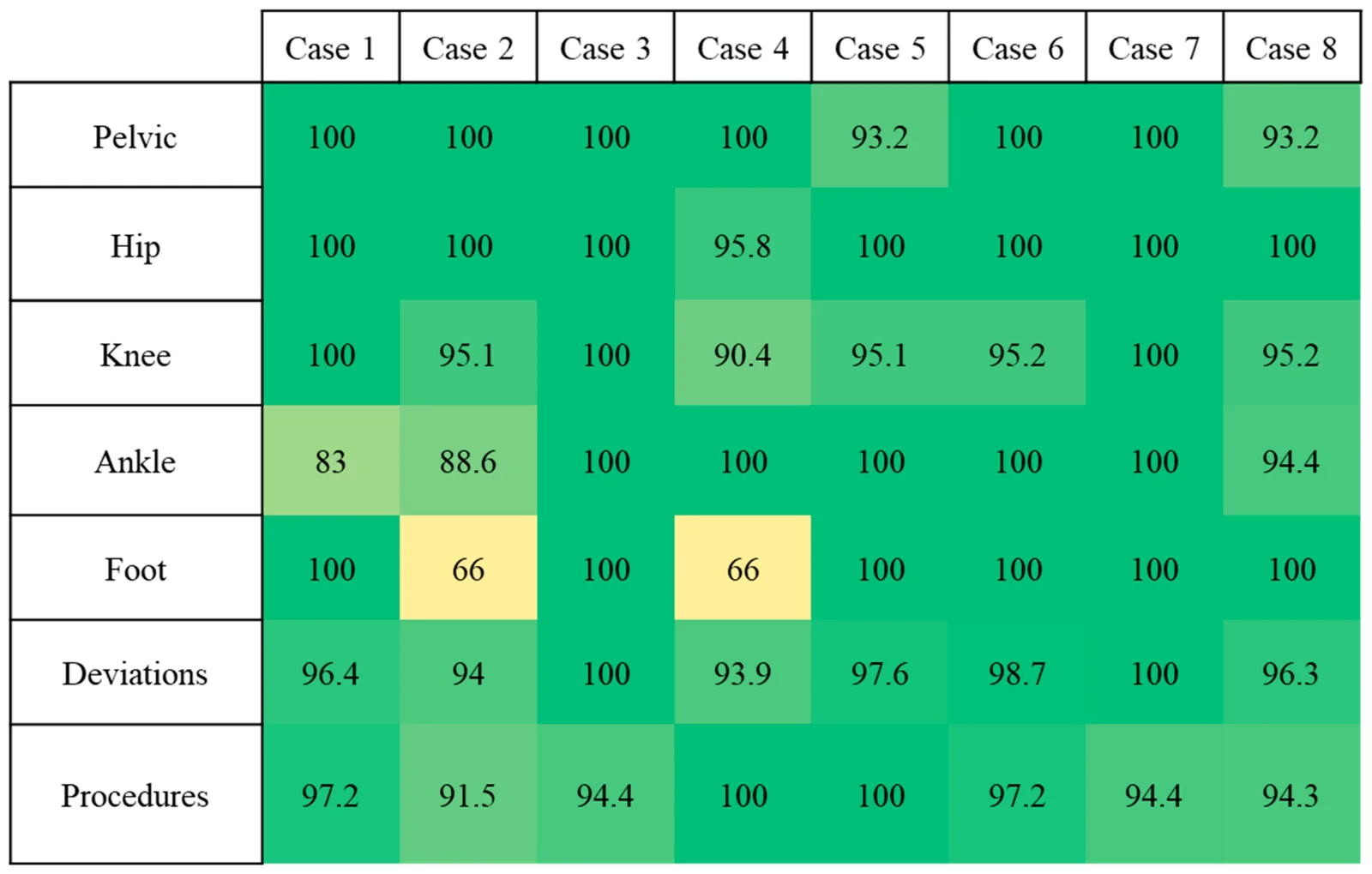
LLM self-consistency (%) across all cases at different segmental levels, averaged over all gait deviations and procedure recommendations. The colour scale, ranging from dark green to yellow, represents the highest (100%) to lowest levels of consistency.

Across all cases, the language model demonstrated an average self-consistency of more than 90% (Figure 3) in detecting deviations and recommending procedures. However, consistency was comparatively lower for foot-level deviations in two cases, where it dropped to 66%.

## 4. Discussion

This pilot study suggests that an LLM-based pipeline driven solely by kinematic features can generate gait deviation assessments and surgical procedure recommendations in children with unilateral CP that substantially align with expert judgement. Although the pipeline demonstrated high internal self-consistency, agreement varied meaningfully across anatomical levels, planes of motion, and procedure groups, while inter-surgeon agreement was only moderate to high. The wide confidence intervals reflect the small number of cases and indicate that these findings should be regarded as preliminary signals, which are discussed in detail in the subsections below.

### 4.1. Gait Deviations

In the sagittal plane, knee flexion and ankle plantarflexion angles at initial contact are two clinically relevant parameters associated with hamstring spasticity [30] and equinus gait pathology [31], respectively. However, these parameters were not included in this study. This omission resulted in partial disagreement (Table 5), suggesting that a more detailed segmentation of the gait cycle beyond the stance and swing phases particularly in the sagittal plane would improve the ability of LLMs to detect and interpret clinically meaningful deviations. The relatively lower agreement observed for knee kinematics in the transverse plane (Table 4) is consistent with known limitations of 3D-IGA in measuring knee rotational angles [32]. Furthermore, the analysis was restricted to the affected side, and the model was unable to evaluate bilateral features such as pelvic obliquity in a comparative context. This limitation made it difficult to distinguish true pelvic deviations from anatomical or functional leg length discrepancies (Table 5), resulting in a reduced agreement in the frontal plane.

As suggested by the gait expert, defining clinically meaningful thresholds relative to reference data could further enhance interpretation. For instance, Baker et al. [33] proposed that deviations exceeding ±1 standard deviation from TD may represent a minimal clinically important difference. Incorporating such thresholds into future implementations could improve the clinical relevance of LLM-based interpretations. Additionally, although reduced knee flexion ROM over the entire gait cycle may be indicative of stiff-knee gait pathology [34], evaluating this parameter across all gait subphases may introduce redundant or clinically non-informative deviations.

The features used in this study have been applied in large-scale machine learning investigations [9,10], yet further research is warranted to optimize their role in interpretability and LLM-based reasoning. Integrating global gait quality indices such as Gait Deviation Index (GDI) [35], may further enhance decision-support performance.

### 4.2. Surgical Procedures

Agreement between the surgical reviewers and the LLM’s reasoning and treatment recommendations varied across the twelve surgical procedure groups. Hip adductor lengthening and selective dorsal rhizotomy showed 100% agreement with both surgeons, whereas tibialis anterior procedures showed lower agreement (31.3% with Surgeon A and 25% with Surgeon B; Table 6). Proximal femoral osteotomy also showed reduced agreement (62.5% and 50%, respectively; Table 6).

The low agreement for tibialis anterior procedures is consistent with the recognized complexity of this clinical decision. In our centre, this intervention is most commonly performed as tibialis anterior tendon shortening combined with Achilles tendon lengthening (TATS-TAL) [36,37]. It is typically indicated for persistent swing-phase drop foot in unilateral CP and requires multimodal clinical information, including EMG and physical examination data, for decision-making, as highlighted by both surgeons (Table 8).

Furthermore, both surgeons reported ambiguity in the presentation of derotation osteotomy and proximal femoral osteotomy as distinct procedure categories. Although derotation osteotomy primarily refers to rotational correction procedures, such as tibial derotation, and is driven by transverse-plane abnormalities, proximal femoral osteotomy addresses varus/valgus deformities associated with frontal-plane deviations. These distinctions, however, were not sufficiently clarified during the review process. This lack of explicit differentiation likely contributed to confusion and represents an additional source of disagreement in procedure selection.

The inclusion of “need more information” (NMI) as a response category provides valuable insight into the model’s reasoning beyond binary decision-making. Comprehensive clinical gait assessment typically integrates multiple sources of information, including passive joint range of motion, muscle function, selectivity, spasticity, patient history, and muscle activity, and kinetic data. Decisions regarding bony procedures often also require imaging data, without which precise recommendations are difficult to establish. Notably, the absence of “YES” outputs for certain procedures combined with the presence of NMI responses supported by appropriate reasoning suggests that LLM was able to appropriately identify gaps in the available clinical information. Importantly, expert raters evaluated both the decision and its underlying reasoning, and in all NMI responses the additional information requested by the LLM was considered clinically relevant (example LLM output in Supplementary Material). Nevertheless, it remains to be determined whether the availability of more complete datasets would enable the model to arrive at accurate and clinically sound decisions.

Additionally, inter-surgeon agreement on procedure recommendations was moderate to high (according to [38]), with an average agreement of 79.2% and a kappa of 0.59 (Table 7). For comparison, Wang et al. [6] reported 84–90% agreement and a treatment-recommendation kappa of 0.59 within a single institution, whereas Rethlefsen et al. [7] observed 66–90% agreement, with modest kappa values of 0.22–0.52, among surgeons from different institutions. In both studies, interpreters had access to comprehensive clinical information, whereas in the current study, only kinematic curves were available. Although our kappa value was comparable to the previously reported single-institution value [6], the wide confidence interval reflects the small sample size of only eight cases. Experience-related differences between the two reviewing surgeons may therefore have contributed substantially to the observed variability.

### 4.3. LLM Self-Consistency

The model demonstrated substantial internal consistency across three independent runs, with average agreement exceeding 90% for both gait deviation detection and procedure recommendation across all eight cases (Figure 3). Only one single decision was classified as INC among 320 total decisions, suggesting that three repeated runs may be sufficient to obtain stable output from generic LLMs. However, it is important to emphasize that high self-consistency does not necessarily imply correctness; a model may produce consistently incorrect outputs, and such consistency can foster a misleading perception of reliability. The employed feature-based approach in this study likely contributed to the high level of self-consistency observed. Nevertheless, the impact of using full gait patterns, rather than extracted features, as input to LLMs warrants further investigation in future work.

It should also be noted that, the total processing time from reading gait data from C3D files through GaitSharing, feature extraction, and API requests to the LLM and output generation was approximately 10–15 min per patient, which is shorter than the duration typically reported for report generation in conventional clinical gait analysis [39]. In addition, our approach may potentially reduce the time required for interpretation and decision-making by providing a detailed report on observed deviations and relevant surgical interventions; however, this variable was not explicitly measured and compared in the current study.

### 4.4. Limitations and Future Directions

This is a single-centre pilot study, and its findings should be interpreted accordingly. The small sample (*n* = 8), restricted to ambulatory children with unilateral CP, limits statistical power and precludes generalisation across CP subtypes, GMFCS levels, age ranges, and complex multi-level involvement; certain CP-related gait pathologies and deformities were therefore not represented. Given this design, formal between-group comparisons across planes of motion, across joints, or between surgeons were not undertaken because such tests would be statistically underpowered and would invite spurious findings. Agreement is reported descriptively as point estimates with 95% CIs; these intervals are necessarily wide, and the per-joint and per-procedure estimates should be interpreted as preliminary signals rather than precise performance values. The comparatively lower agreement observed for some planes, joints, and procedures may therefore partly reflect sampling variability rather than true performance differences, and the findings are best regarded as hypothesis-generating, requiring confirmation in an adequately powered, multi-centre study with prospectively defined subgroups.

CP is also a heterogeneous clinical condition, and its management is not confined to surgical interventions alone. Non-surgical approaches, e.g., physiotherapy, occupational therapy, and the use of orthotics and assistive devices, play a crucial role in treatment planning, and future investigations should evaluate LLM-based decision support in more comprehensive clinical contexts. In the current study, other clinical signals such as EMG and ground reaction forces were excluded from the analysis due to their inherent redundancy with the kinematic features. Nevertheless, even with kinematic data alone, agreement between surgeons and the LLM (78.7%) was comparable to inter-surgeon agreement (79.2%). This suggests that the kinematic feature set captures a substantial portion of the information used by surgeons when reviewing gait data in isolation.

The influence of clinical experience on agreement with the LLM was not a research question in this study, and the review process was kept anonymised accordingly. This factor could be investigated in future work by involving a larger and more diverse cohort of surgeons. The gait deviation assessment would similarly benefit from independent rating by two or more gait analysis experts to strengthen the reliability analysis; this was beyond the scope of the present study, in which the primary focus was on the final surgical recommendation level. Future studies should include multiple experts from different centers to assess inter-rater reliability and enhance the reliability of gait deviation detection results.

The integration of probabilistic decision-support tools such as EB-GAIT [8], developed on local clinical data, with natural language reasoning systems trained on publicly available knowledge and scientific literature, represents a promising direction for the next generation of clinical gait analysis decision support.

Locally deployed LLMs, augmented with retrieval over institution-specific reports, imaging notes, EMG, and outcome data, would allow each centre to keep patient data on-site while still benefiting from accumulated expertise, as an approach that may be especially valuable for smaller centres that lack decades of in-house gait-analysis experience but could draw on the experience embedded in larger reference centres. Multi-centre studies built on shared data standards and a common open toolkit would in turn allow systematic comparison of LLM performance across institutions and CP subtypes, and extension of the framework to other gait pathologies such as stroke and Parkinson’s disease. Finally, the development of time-series foundation models for gait analysis, operating directly on full kinematic waveforms rather than extracted features, could move such systems beyond reproducing expert interpretations toward identifying the underlying locomotor strategies that organise the observed gait pattern.

## 5. Conclusions

In conclusion, this pilot study highlights the potential of generative LLMs as supportive tools in clinical gait analysis, particularly for detecting gait deviations and informing future treatment planning decisions. We introduced a practical, end-to-end pipeline, accompanied by the open-source and freely available GaitSharing toolkit [25,26], to encourage further research on the role of LLMs in clinical decision support. Despite the limited sample size, the single-centre design, and the exclusive use of kinematic data, the proposed framework showed substantial but variable agreement with expert assessment for both gait deviation interpretation and surgical procedure recommendations, alongside high LLM self-consistency (>90% across runs). Beyond these preliminary performance estimates, the broader value of this work may lie in providing a consistent, transparent, and evidence-linked starting point for gait interpretation, particularly for centres without access to decades of accumulated gait-analysis experience. Realising that potential will require adequately powered, multi-centre evaluation, ideally combining locally deployed models with retrieval over institution-specific clinical data, before such systems can be considered for routine clinical use.

## Figures and Tables

**Table 1:** Group details. Uni-CP: children with unilateral cerebral palsy, TD: typically developing children, M: male, F: female, R: right, L: left, NA: not applicable.

Group	Parameter					
	N	Sex	Age (Years)	Height (m)	Weight (kg)	Affected Side
		M/F	Mean [Range]	Mean [SD]	Mean [SD]	R/L
Uni-CP	8	6 M/2 F	10.3 [7.2–14.8]	1.39 [0.15]	36.4 [19.9]	4 R/4 L
TD	20	10 M/10 F	11.4 [7.6–16.7]	1.47 [0.18]	37.3 [12.0]	NA

**Table 2:** Predefined kinematic gait deviations assessed by the LLM in Phase 1. ROM: range of motion.

Joint	Deviation
Pelvis	Increased anterior pelvic tilt
	Reduced pelvic sagittal ROM
	Pelvic obliquity in stance
	Pelvic rotation asymmetry
	Excessive pelvic rotation
Hip	Excessive hip flexion in stance
	Reduced peak hip extension in stance
	Reduced hip sagittal ROM
	Excessive hip adduction in stance
	Excessive hip adduction in swing
	Excessive hip abduction in stance
	Internal hip rotation in stance
	External hip rotation in stance
Knee	Excessive knee flexion in stance
	Knee hyperextension in stance
	Reduced peak knee flexion in swing
	Delayed timing of peak knee flexion in swing
	Reduced knee sagittal ROM
	Internal knee rotation in stance
	External knee rotation in stance
Ankle	Reduced peak ankle dorsiflexion in stance
	Excessive peak ankle dorsiflexion in stance
	Excessive ankle plantarflexion in swing
	Reduced ankle sagittal ROM
	Ankle valgus in stance
	Ankle varus in stance
Foot	Foot internal rotation
	Foot external rotation

**Table 3:** Predefined surgical procedure groups assessed by the LLM in Phase 2.

No.	Procedure Group
1	Calf muscle lengthening
2	Tibialis anterior procedure
3	Tibialis posterior procedure
4	Foot bony reconstruction
5	Hamstring lengthening
6	Rectus femoris transfer
7	Distal knee surgery
8	Derotation osteotomy
9	Hip adductor lengthening
10	Psoas procedure
11	Proximal femoral osteotomy
12	Selective dorsal rhizotomy

**Table 4:** Agreement between the gait expert and the LLM outputs, stratified by anatomical level and plane of motion. Values are presented as percentage agreement with 95% confidence intervals (CIs). Agreements below 75% are highlighted in red. NA: not applicable.

	Sagittal (95% CI)	Frontal (95% CI)	Transverse (95% CI)	Overall (95% CI)
Pelvic	87.5 (68.8–100)	62.5 (25–87.5)	100 (100–100)	87.5 (75–97.5)
Hip	91.7 (79.2–100)	91.7 (83.3–100)	87.5 (68.8–100)	90.6 (82.8–96.9)
Knee	68.8 (55–82.5)	NA	56.3 (50–68.8)	65.2 (54.5–75)
Ankle and Foot	90.6 (82.8–96.9)	62.5 (50–81.3)	100 (100–100)	85.9 (81.3–90.6)
Overall	82.6 (75–90.2)	77.1 (68.8–85.4)	85.9 (81.3–90.6)	82.4 (77–87.5)

**Table 5:** Overview of gait expert feedback outlining the observed concerns regarding the LLM outputs and the methodology. ROM: range of motion, IC: initial contact.

Concern	Gait Expert Feedback
Clinically relevant	The model should be informed of a clinically relevant difference threshold (e.g., 1.5 standard deviation from healthy reference data) to distinguish pathological deviations from normal variability.
Phases ROM	Range of motion should be computed over the whole gait cycle only; reporting ROM for stance and swing phases separately is unnecessary and may confuse interpretation. Whole-cycle ROM is most relevant for the knee (stiff knee detection) and pelvic (increased ROM).
Pelvic ROM	Pelvic ROM is typically stable across healthy; therefore, an increased ROM should also be considered a deviation, not a reduced ROM.
Pelvic obliquity	Pelvic obliquity and rotation asymmetry requires comparison of both left and right gait cycles to distinguish anatomical or functional leg length discrepancy from a true pelvic deviation. Assessing the affected side alone may be insufficient.
Knee flexion at IC	Knee flexion angle at IC is an important indicator for downstream decision-making, particularly regarding orthotic prescription, and was not captured.
Ankle angle at IC	Ankle angle at IC is clinically important for identifying flat/fore-foot contact patterns (initial flat foot or forefoot contact), which was not included in the deviation list.
Ankle valgus/varus	Valgus and varus as measured during gait represent the alignment between the tibial segment and the global laboratory axis, not ankle joint angle. This should be acknowledged when interpreting these items. More accurate information can come from clinical examinations and clinical images.
Foot rotation	“Foot internal/external rotation” in the prompt should be renamed to “internal/external foot progression angle,” as the foot rotation signal from marker-based gait analysis is not a reliable measure of true foot rotation.

**Table 6:** Agreement between each surgeon and the LLM outputs across all cases, stratified by surgical procedure group. Values are presented as percentage agreement with 95% confidence intervals (CIs). Agreements below 75% are highlighted in red.

Procedure Group	Agreement of Surgeon A on LLM Across All Cases (95% CI)	Agreement of Surgeon B on LLM Across All Cases (95% CI)
Calf muscle lengthening	75 (37.5–100)	75 (37.5–100)
Tibialis anterior procedure	31.3 (6.3–62.5)	25 (0–50.3)
Tibialis posterior procedure	87.5 (62.5–100)	75 (37.5–100)
Foot bony reconstruction	75 (37.5–100)	68.8 (37.5–100)
Hamstring lengthening	93.8 (81.3–100)	87.5 (62.5–100)
Rectus femoris transfer	100 (100–100)	68.8 (43.8–93.8)
Distal knee surgery	93.8 (81.3–100)	100 (100–100)
Derotation osteotomy	87.5 (68.8–100)	81.3 (56.3–100)
Hip adductor lengthening	100 (100–100)	100 (100–100)
Psoas procedure	100 (100–100)	50 (12.5–87.5)
Proximal femoral osteotomy	62.5 (25–87.5)	50 (12.5–87.5)
Selective dorsal rhizotomy	100 (100–100)	100 (100–100)
Average	83.9 (78.1–89.1)	73.4 (67.7–79.2)

**Table 7:** Inter-surgeon agreement between the two surgeons for each patient. The average agreement with 95% confidence intervals (CIs) and quadratic-weighted Cohen’s kappa. Agreements below 75% are highlighted in red.

Patients	Inter-Surgeon Agreement (95% CI)
Case 1	83.4
Case 2	75
Case 3	83.4
Case 4	91.7
Case 5	83.4
Case 6	83.4
Case 7	75
Case 8	58.3
Average	79.2 (71.9–85.4)
Cohen kappa	0.59 (0.47–0.7)

**Table 8:** Case-wise qualitative comments on the LLM-generated outputs for surgery procedures, provided by surgeons A and B. TAL-TATS: Tendo-Achilles lengthening with tibialis anterior tendon shortening, EMG: electromyography.

Case	Surgeon A	Surgeon B
1	This patient is a candidate for the TAL-TATS procedure. An additional assessment for possible bony foot correction is recommended.	Would proceed with calf lengthening if a contracture is confirmed and further consider tibialis anterior shortening based on EMG results. If assessments indicate flatfoot, a bony correction may be needed. Psoas lengthening is not recommended due to the patient’s good function and risk of muscle weakening.
2	A knee extension osteotomy should be considered. Hip flexor contractures should be assessed to determine whether lengthening is needed.	Need additional information on the patient’s muscle strength, EMG findings, contractures, and foot deformity before planning for surgical treatment.
3	candidate for TAL-TATS. Torsional alignment should be assessed through rotational range and femoral anteversion. A derotation osteotomy is indicated when internal rotation predominates with limited external rotation and increased anteversion; if external rotation is good, the issue is likely muscle weakness, and no bony correction is needed.	Depending on the clinical data, including clinical assessment, video analysis, and the torsional profile, it is likely to consider tibialis anterior shortening, calf lengthening, and hip derotation. The patient’s age may influence the decision regarding knee valgus correction; in boys between approximately 12–14 years and girls between about 10–12 years, such correction may be considered.
4	Distal correction is recommended, including calf muscle lengthening and procedures involving the tibialis anterior and posterior. Torsional malalignment should also be evaluated.	Would likely proceed with calf lengthening and tibialis anterior shortening, based on clinical assessment and EMG findings. Hip derotation with foot correction may also be considered depending on torsional values, foot imaging, and kinetic data.
5	Equinus correction should be performed together with proximal bony realignment. Rectus femoris intervention should also be considered; botulinum toxin injection may serve as an alternative.	Calf lengthening, tibialis anterior shortening, and hip derotation appear to be the most plausible options, but additional clinical data is required before making a final decision.
6	This patient requires drop foot correction, with a possible tibialis anterior procedure.	A foot deformity seems more likely in this case, and we need additional information at this level to make an informed surgical recommendation. This includes foot imaging and clinical assessment of the calf muscles to better understand muscle function and potential contracture.
7	Equinus correction is indicated together with a tibialis anterior procedure. Tibial torsional alignment should be verified through clinical examination.	The patient appears to be well-compensated proximally, but at the distal level we may need to consider equinus correction, including tibialis anterior shortening, depending on clinical/EMG.
8	Clinical evaluation of knee and hip function is recommended prior to any surgical planning. No distal surgical intervention is indicated at this stage. An assessment of the torsional profile is required.	The patient shows good distal function, but more details on foot alignment and ankle range of motion are needed. The significance of observed hip and knee external rotation remains unclear. The unaffected side demonstrates compensatory gait patterns, and all these factors must be clarified before making decision.

## Data Availability

The full GaitSharing toolkit, including the LLM Interpreter module, the structured prompt used in this study, and all processing scripts, is openly available under MIT License at [25,26]. The de-identified feature files and the corresponding LLM reports analyzed in this study are available from the corresponding author upon reasonable request, subject to the institutional data-sharing policy of the University Children’s Hospital Basel.

## Supplementary Materials

The following supporting information can be downloaded at: https://www.mdpi.com/article/10.3390/bioengineering13080862/s1. The full prompt text (StudyPrompt.txt), together with an example LLM input (LLMInput_example.txt; kinematic features) and output (LLMOutput_example.xlsx; procedure recommendation), is provided in the Supplementary Material. Detailed data underlying Figure 1 are available in DetailedTable_Figure1.xlsx.

## Abbreviations

The following abbreviations are used in this manuscript:

CP	Cerebral palsy
3D-IGA	Three-dimensional instrumented gait analysis
LLM	Large language model
AI	Artificial intelligence
EMG	Electromyography
ROM	Range of motion
EB-GAIT	Evidence-Based Gait Analysis Interpretation Tools
ChatGPT	Chat Generative Pre-trained Transformer
API	Application Programming Interface
NMI	Need more information
INC	Inconclusive
TAL-TATS	Tendo-Achilles lengthening with tibialis anterior tendon shortening
IC	Initial contact
GDI	Gait Deviation Index
